# Hypnotism or Mesmerism

**Published:** 1889-11-02

**Authors:** J. A. Manton

**Affiliations:** Demonstrator of Anatomy, Firth College, Sheffield


### Hypnotism or Mesmerism.

By J. A. Manton, M.R.C.S., Eng., L.R.C.P., Lond., Demonstrator of Anatomy, Firth College, Sheffield.

The interest taken in psychological problems nowadays has caused attention to be again drawn to the once much-discussed subject of hypnotism. Now that the lay press have taken up the matter all sorts of wild ideas are prevalent concerning it. Possibly the experiences and convictions of a medical man who possesses this so-called gift of hypnotism or mesmerism in an eminent degree, may be of interest to many readers. In a recent issue of The Hospital we are pertinently asked whether mesmerism is of any practical utility in the treatment of diseases. As an unbiassed and, I trust, honest investigator, I am bound to say that the cases where it can be usefully employed are few and far between. It is impossible that mesmerism can be of general utility, for the simple reason that susceptibility to its influence is the exception, and not the rule. In fact, some eminent authorities go so far as to say that anyone possessing the *mens sana in corpore sano* cannot be influenced by hypnotism—*i.e.*, susceptibility to its influence must be looked upon as indicative of a morbid condition of the cerebrum and its surroundings. This statement is partly borne out by the fact that the mesmerist numbers amongst his most suitable subjects persons of a "neurotic" type, hysterical girls and strumous boys budding into adolescence. In fact, about the first thing that one notices at public entertainments in which mesmerism is a feature is an absence of a look of "intellectuality" on the faces of the subjects. Bright, intelligent faces are, to use a hackneyed phrase, "conspicuous by their absence." People have an idea that mesmerism is a sort of mental chloroform that can be turned on and off at the will of the operator. To correct a popular error, it may be as well to say that no one can succumb to its influence against their will. I have examined a large number of "sensitives," and have come to the conclusion that they can be roughly classified as follows:

(*a*) Fair strumous girls of a *spirituelle* type, generally hysterical.

(*b*) Young girls of the greasy, dark strumous type.

(*c*) Persons of epileptic tendency, with low foreheads, prominent noses (*a la* Lefroy), thick lips, and retreating chins.

(*d*) Many imbeciles and people of weak intellect are singularly susceptible. Possibly this may account for the wonderful stories about the power of the human eye, and also the faculty some physicians have of quelling refractory lunatics with a glance.

Many public performers—honest men without doubt—are under the impression that they are endowed with power not granted to ordinary individuals. Anyone possessed of a strong will, steady gaze, robust health, and last, but not least, a stern or grave cast of countenance (a jovial-looking experimentalist would stand very little chance of being successful) can produce most of the startling effects seen on the platform. "How's it done?" I almost fancy I hear some of my readers exclaim. The following is the method I usually employ. Having obtained a suitable and willing subject, place him in a chair before you and tell him to cultivate an attitude of complete mental passivity. Assure him that there is no danger, and try to impress him with an idea of your wonderful powers. Speak slowly and solemnly. Gaze at his eyes fixedly. If there be any tendency to giggling or restlessness, hold a bright metal disc in front and above the sitter's head, so that in fixedly gazing at it he is obliged to slightly strain his eyes. Allow him to do this for a few moments, then let your own eyes replace the disc, continue the gaze for a few minutes, then gently close his eyes and concentrate your whole will on the idea of closing the eyelids tightly. Press the thumbs over the supra-orbital foramen, gradually increasing the pressure. Repeat this a few times, and then say, in a low, commanding voice, "You can't open your eyes," willing strongly that he shall not. You will find that he is, in many cases, unable to do so. Should he disappoint you, repeat the process, and, sooner or later, you are almost certain to succeed. Having closed his eyes, you can tell him to go to sleep, and to sleep he will go. From this slumber he will waken naturally in a period varying from one half to twenty-four hours, or immediately on the operator giving him a slap on the shoulder, or blowing in his face, exclaiming at the same time, "Wake up!" or any similar ejaculation. Just after the closure of the sitter's eyelids will be found the most suitable time for experiment. If you now give your subject a piece of stick and tell him that it is a flute and bid him play, he will forthwith proceed to operate on this imaginary instrument, and if you awaken him will probably be covered with confusion at seeing the ludicrous antics that he has been engaged in.

In the deeper stages of hypnotism there is insensitiveness of the eye, insensibility to pain, and in rare cases complete insensibility. I find, however, "sensitives" which are quite proof against an *expected* pain, frequently flinch violently at an *unexpected* one. A scientific friend of mine caused an American seance to break up in uproar and confusion by trying the somewhat novel experiment of putting a pin under the "sleeping beauty's" nail. Beauty fled "'midst hideous ruin and combustion." The influence of will in hypnotism is a puzzling crux to the investigator. Mesmerists assert that will is a force and can be transferred from one person to another, and that this is responsible for "love at first sight," and a host of kindred phenomena; but alas! for this theory, Dr. Braid, of Manchester, the "hypnotic heretic," proved that the bulk of mesmeric phenomena could be produced without the intervention of a mesmerist, and by simply using the metallic disc above alluded to. In reply to this the mesmerists say "Oh! but we can mesmerise a man without catching his eyes at all," the *modus operandi* being as follows: The subject is placed with his back to the mesmerist, who *wills* that he shall fall, and backward he falls—he is *expected* to fall, and he does fall. I am willing to admit that this effect may be the result of expectancy and self-delusion, but I do not think we are justified in attributing it to "pyschic," "odic," or any other force. Hypnotism is a most unreliable therapeutic agent. 'Tis true that tongues and ears have been pierced during the mental stupor caused by this influence, and even arms and legs have occasionally been amputated, but these cases, as stated at the beginning of my article, are very rare. Headaches, neuralgia, etc., often disappear under the influence of "passes," but as these ailments often belong to the order of *malades imaginaires*, too much importance must not be attached to the fact. It is stated that "cancers" can be thus caused to disappear, but most practitioners will take this with a very liberal allowance of salt. I once, however, came across a case where "passes" caused the disappearance of a fatty tumour in the upper portion of the arm. I am honestly bound to admit that cases of epilepsy seem to be greatly benefited by this odd method of treatment.

Mesmerism in some of its aspect bears a great resemblance to the much vaunted but, alas! unsatisfactory method of "faith healing." The whole subject, however, is well worthy of serious investigation by competent authorities. One earnest final caution I would give to all concerned in tending the sick, whether doctors, students, or nurses, and that is, "Don't experiment on your patients."

